# Lowered expression of microRNA-125a-5p in human hepatocellular carcinoma and up-regulation of its oncogenic targets sirtuin-7, matrix metalloproteinase-11, and c-Raf

**DOI:** 10.18632/oncotarget.15809

**Published:** 2017-02-28

**Authors:** Nicola Coppola, Giorgio de Stefano, Marta Panella, Lorenzo Onorato, Valentina Iodice, Carmine Minichini, Nicola Mosca, Luisa Desiato, Nunzia Farella, Mario Starace, Giulia Liorre, Nicoletta Potenza, Evangelista Sagnelli, Aniello Russo

**Affiliations:** ^1^ Department of Mental Health and Public Medicine, Second University of Naples, Naples, Italy; ^2^ IX Interventional Ultrasound Unit for Infectious Diseases, AORN dei Colli, P.O. Cotugno, Naples, Italy; ^3^ Department of Environmental, Biological and Pharmaceutical Sciences and Technologies, Second University of Naples, Naples, Italy

**Keywords:** microRNA, gene regulation, cell proliferation, tumor, hepatocellular carcinoma

## Abstract

Human microRNA-125a-5p (miR-125a) is expressed in most tissues where it downregulates the expression of membrane receptors or intracellular transductors of mitogenic signals, thus limiting cell proliferation. Expression of this miRNA generally increases with cell differentiation whereas it is downregulated in several types of tumors, such as breast, lung, ovarian, gastric, colon, and cervical cancers, neuroblastoma, medulloblastoma, glioblastoma, and retinoblastoma. In this study, we focused on hepatocellular carcinoma and used real-time quantitative PCR to measure miR-125a expression in 55 tumor biopsies and in matched adjacent non-tumor liver tissues. This analysis showed a downregulation of miR-125a in 80 % of patients, with a mean decrease of 4.7-fold. Comparison of miRNA downregulation with clinicopathological parameters of patients didn't yield significant correlations except for serum bilirubin. We then evaluated the expression of known targets of miR-125a and found that sirtuin-7, matrix metalloproteinase-11, and c-Raf were up-regulated in tumor tissue by 2.2-, 3-, and 1.7-fold, respectively. Overall, these data support a tumor suppressor role for miR-125a and encourage further studies aimed at the comprehension of the molecular mechanisms governing its expression, eventually leading to treatments to restore its expression in tumor cells.

## INTRODUCTION

Primary liver cancer is the fifth most common cancer in men and the ninth in women, with 554,000 and 228,000 new cases per year, respectively. In addition, it is the second most common cause of death for cancer worldwide, estimated to be responsible for nearly 750,000 deaths per year, i.e., 9% of all cancer deaths [1]. Hepatocellular carcinoma (HCC) is the predominant type of primary liver cancer and arises mostly in cirrhotic livers [2]. Since a few therapeutic options exist, particularly in the more advanced stages that require a systemic treatment, there is an urgent need for new biomolecular markers for an early diagnosis and of new therapeutic targets to improve the survival rate of patients with HCC.

MicroRNAs (miRNAs) are small non-coding RNAs that negatively regulate gene expression at post-transcriptional level by affecting both translation and stability of complementary mRNAs [3]. MiRNAs are involved in a large variety of physiological processes playing crucial roles in cell differentiation and development [4, 5]. Several studies have also shown that aberrant expression of miRNAs is linked to pathological conditions, including cancer [6]. Downregulation of the microRNA biosynthesis enzyme Dicer in cancer cells or mutations affecting its structure lead to dysregulated miRNA biogenesis and increased tumor progression [7–10]. On the other hand, differentiating cells often exhibit increased Dicer expression [11–13]. In this field, a growing body of evidence indicates that miRNAs can function as either tumor suppressors by down-regulating oncogenic proteins, or tumor promoters by limiting the expression of oncosuppressor genes [14–16]. In HCC, miR-122 and miR-199 are frequently downregulated, suggesting a tumor suppressor role whereas miR-21 and miR-221 are often hyperexpressed [17–19]. Sorafenib is the only therapeutic agent for treatment of advanced HCC [20, 21] and a recent study has shown that miR-125a is a downstream effector of the drug in its antiproliferative activity toward carcinoma cells [22]; other microRNAs may also be involved in the mechanism of action of the drug [23].

MiR-125a regulates the expression of several genes controlling cell proliferation, migration, and apoptosis [24]. In the human breast cancer cell line SKBR3, tyrosin kinase receptors ERBB2 and ERBB3 are downregulated by miR-125a leading to diminished cell proliferation and migration [25]. This finding is corroborated by *in vivo* studies showing that miR-125a and -125b are downregulated in ERBB2-amplified and ERBB2-overexpressing breast cancers [26]. In several breast cancer cell lines, miR-125a also targets HuR, an RNA-binding protein that stabilizes transcripts of genes regulating cell proliferation, angiogenesis, rapid inflammatory response and stress response [27]. Overall, these data indicate that miR-125a may counteract proliferation and invasion of breast cancer cells through the downregulation of ERBB2, ERBB3, and/or HuR. These conclusions are also supported by the discovery of a germline mutation in the sequence of mature miR-125a that is highly associated with development of breast cancer [28].

A tumor suppressive role for miR-125a is also supported by a study performed in the human neuroblastoma SK-N-BE cell line [29]. MiR-125a was found to downregulate the truncated isoform of the neurotrophin receptor tropomyosin-related kinase C (t-trkC), with subsequent inhibition of cell proliferation. This finding is consistent with the observed underexpression of miR-125a in human primary neuroblastomas. MiR-125a is also down-regulated in other tumors, such as medulloblastoma [30], glioblastoma [31], and lung cancer [32] where it suppresses cell proliferation by targeting Zbtb7a proto-oncogene [33].

In HCC Hep3B and SNU-449 cells, miR-125a inhibits cell proliferation through the down-regulation of sirtuin-7 (SIRT7), a NAD(+)-dependent deacetylase, and subsequent p21-dependent cell cycle arrest in G1 [34]. This activity has recently been confirmed in HepG2 and HuH-7 cells [22]. In HCC, miR-125a is also known to target vascular endothelial growth factor A (VEGF-A), and matrix metalloproteinase-11 (MMP11) [35].

In this study, we contributed to the characterization of the tumor suppressive activity of miR-125a by measuring its expression in HCC biopsies and in matched adjacent non-tumor liver tissues and by correlating the obtained data with clinical presentation. The expression levels of validated targets of miR-125a were also determined.

## RESULTS

### Characteristics of patients

At the end of the enrollment period, 55 consecutive patients were included in the study. Their demographic, biochemical, virological, and clinical characteristics are summarized in Table 1. The mean age was 70.3 years, 32 (58.1%) patients were males, and 41 (74.5%) were anti-HCV positive with 33 (80.5%) of them showing detectable plasma HCV-RNA. HBV was identified as the etiologic agent of the disease in 10 (17.5%) patients and the remaining 4 (7.3%) had a NASH-related cirrhosis. Two patients had a history of alcohol abuse, both anti-HCV/HCV RNA positive; no other patients had a multiple etiology. Most of patients showed a compensated liver disease, with a Child-Pugh class-A in 89.1% of cases and classes-B/C in 10.9%. 27 (49.1%) patients showed an unifocal and 28 (50.9%) a multifocal HCC. According to the Barcelona Clinic Liver Cancer (BCLC) class, 47 (85.4%) had class A, 5 (9.1%) class B and 3 (5.5%) class C. Three patients showed also portal thrombosis.

**Table 1:** Demographic, biochemical, virological, and clinical characteristics of the enrolled patients

**N° patients**	55
**Mean age (±SD)**	70.3 (6.0)
**Males, n° (%)**	32 (58.1)
**Alcohol abusers (> 30g/die), n° (%)**	2 (3.6)
**BMI (mean ± SD)**	26.8 (3.1)
**Subjects with diabetes, n° (%)**	7 (12.7)
**AST/ULN(mean ± SD)**	1.54 (0.9)
**ALT/ULN (mean ± SD)**	1.49 (0.9)
**ALP/ULN (mean ± SD)**	1.12 (0.6)
**Total bilirubin, mg/dl (mean ± SD)**	0.98 (0.5)
**PT% (mean ± SD)**	89.2 (12.8)
**α-fetoprotein, (mean ± SD)**	233.8 (713.4)
**Anti-HCV-positive patients, n° (%)**	41 (74.5)
- **HCV-RNA-positive subjects, n° (%)**	33 (80.5)
- **HCV load, IU/mL (mean ± SD)**	1.2 E+6 (1.68 E+6)
- **with HCV-genotype 1, n° (%)**	28 (68.3)
- **with HCV-genotype non-1, n° (%)**	6 (14.6)
**HBsAg-positive patients, n° (%)**	10 (17.5)
- **HBV-DNA-positive subjects, n° (%)**	3 (30.0)
- **HBV load, IU/mL (mean ± SD)**	1.13 E+4 (1.91 E+4)
**Patients with NASH, n° (%)**	4 (7.3)
**Child Pugh score, n° (%) of patients with**	55
- **A**	49 (89.1)
- **B**	6 (10.9)
- **C**	0 (0.0)
**Patients with first diagnosis of HCC, n° (%)**	36 (65.5)
**Patients with HCC relapse, n° (%)**	19 (34.5)
**Patients with a single HCC, n° (%)**	27 (49.1)
**Patients with multiple HCC, n° (%)**	28 (50.9)
**Patients with portal thrombosis, n° (%)**	3 (5.4)
**BCLC score, n° (%) of patients with**	55
- **A**	47 (85.4)
- **B**	5 (9.1)
- **C**	3 (5.5)

### Down-regulation of miR-125a in hepatocellular carcinoma

**Figure 1:**
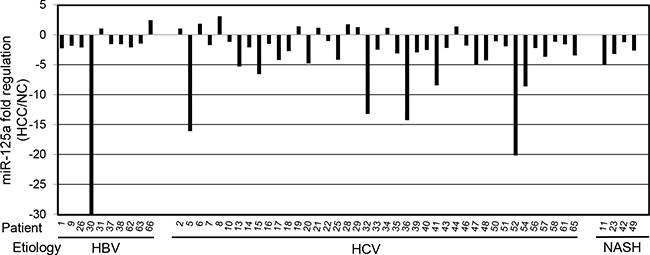
Expression of miR-125a in hepatocellular carcinoma biopsies from 55 patients MiR-125a was quantitated by RT-qPCR in biopsies of hepatocellular carcinoma (HCC) from patients with viral hepatitis (HBV and HCV) or non-alcoholic steatotic hepatitis (NASH). For each patient, the expression level is reported as fold regulation for comparison with matched adjacent non-tumor liver tissue (NC). The out of range value for patient 30 is -94.

**Figure 2:**
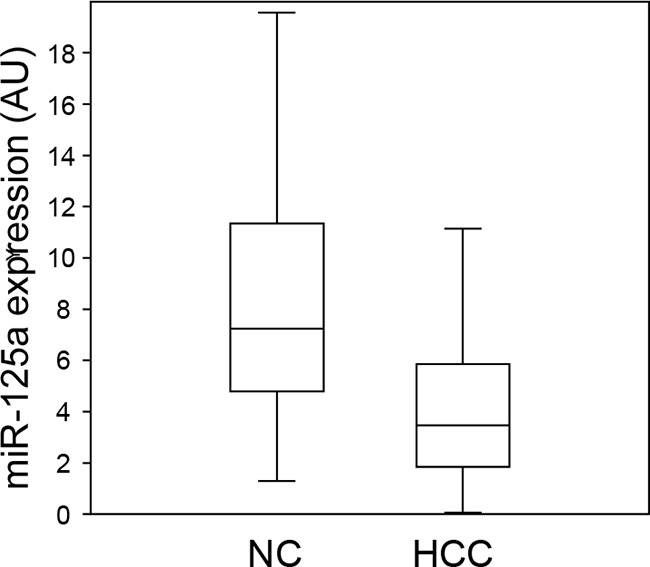
Comparison of the expression levels of miR-125a in hepatocellular carcinoma and non-tumor liver tissue Each box plot depicts data from 55 patients. The vertical lines indicate the value ranges, the horizontal boundaries of the boxes represent the first and third quartile. The p value for the comparison of the two data sets is <0.0000001 at Student's t-test.

**Figure 3:**
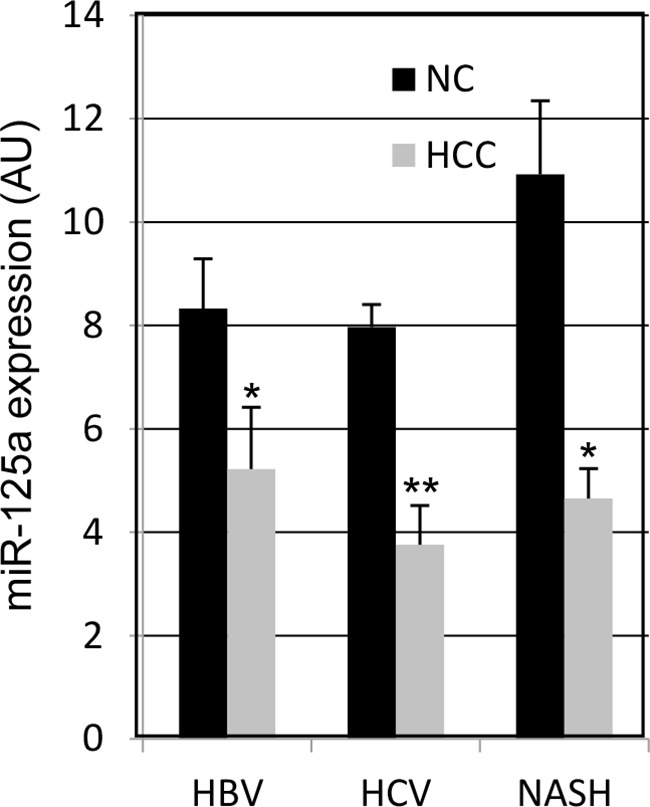
MicroRNA-125a expression in HCC from patients with viral or non-alcoholic steatotic hepatitis Data are the mean ± SEM. * p < 0.05 and ** p < 0.00001 at Student's t-test for comparison with NC tissue.

Real-time qPCR was used to measure the amount of miR-125a in HCC biopsies from 55 patients with viral hepatitis or NASH. Comparison with the adjacent non-tumor liver tissue (NC) revealed a down-regulation of the miRNA in 44 out of 55 patients (80%) (Figure 1), with a mean decrease of 4.7-fold. When the absolute amounts of the miRNA were considered, the mean content in HCC was 48% of that in NC and difference was highly significant (Figure 2). When patients were grouped based on HCC etiology, downregulation of the miRNA was detected in 8 out of 10 HBV patients (80%), 32 out of 41 HCV patients (78%), and 4 out of 4 patients with NASH. Down-regulation was statistically significant in each group and prominent in HCV patients whose expression of miR-125a was reduced to 45 % (Figure 3). This result was confirmed even when HCV-RNA positive (3.83±3.14 vs. 7.39±4.64 AU, p=0.0005) and HCV-RNA negative patients (4.78±3.81 vs. 10.3±5.01 AU, p=0.027) were separately analyzed.

### Correlation between down-regulation of miR-125a in HCC and clinical characteristics

One of the aims of the study was to correlate the expression profile of miR-125a-5p in neoplastic and non neoplastic tissue and the clinical characteristics of the subjects enrolled. When patients were stratified according to age, gender, transaminase levels, Child-Pugh score, BCLC score or other clinical characteristics of HCC (Table 2), no difference was observed between the mean fold-regulation of the miRNA in HCC vs. NC tissue. However, patients with total bilirubin serum concentrations higher than 1,1 mg/dL showed a markedly lower fold-regulation (-6.72±6.5 vs -1.84±2.63, p=0.02)] of the miRNA and no patient had a Gilbert's syndrome.

**Table 2:** Correlation between miRNA expression and demographic, biochemical, virological, and clinical characteristics of the patients

	HCC vs. NC fold-regulation ≤ -2.13	HCC vs. NC fold-regulation > -2.13	p
**N° patients**	28	27	
**Mean age (±SD)**	70.2 (5.6)	70.3 (6.6)	0.96
**Males, n° (%)**	14 (50.0)	18 (66.7)	0.21
**Alcohol abusers (> 30g/die), n° (%)**	0 (0.0)	2 (7.4)	0.14
**BMI (mean ± SD)**	26.2 (2.8)	27.6 (3.4)	0.15
**Subjects with diabetes, n° (%)**	4 (14.3)	3 (11.1)	0.99
**AST/ULN(mean ± SD)**	1.47 (0.9)	1.61 (0.9)	0.60
**ALT/ULN (mean ± SD)**	1.45 (0.9)	1.54 (0.9)	0.72
**ALP/ULN (mean ± SD)**	1.1 (0.3)	1.2 (0.8)	0.47
**Total bilirubin, mg/dl (mean ± SD)**	1.17 (0.6)	0.78 (0.3)	0.008
**PT% (mean ± SD)**	88.2 (12.6)	90.3 (13.1)	0.55
**α-fetoprotein, (mean ± SD)**	119.9 (413.8)	363.7 (941.4)	0.27
**Anti-HCV-positive patients, n° (%)**	22 (78.6)	19 (70.4)	0.48
- **HCV-RNA-positive subjects, n° (%)**	18 (81.8)	15 (78.9)	0.81
- **HCV load, IU/mL (mean ± SD)**	1.02 E+6 (1.53 E+6)	1.42 E+6 (1.87 E+6)	0.51
- **with HCV-genotype 1, n° (%)**	17 (77.3)	11 (57.9)	0.18
**HBsAg-positive patients, n° (%)**	3 (10.7)	7 (24.1)	0.14
- **HBV-DNA-positive subjects, n° (%)**	1 (30.0)	2 (28.6)	0.88
- **HBV load, IU/mL (mean ± SD)**	3.34 E+4	3.06 E+2 (5.1 E+1)	NA
**Patients with NASH, n° (%)**	3 (10.7)	1 (3.7)	0.32
**Child Pugh score, n° (%) of patients with**			
- **A**	24 (85.7)	25 (92.6)	
- **B**	4 (14.3)	2 (7.4)	
- **C**	0 (0.0)	0 (0)	0.41
**Patients with first diagnosis of HCC, n° (%)**	19 (67.9)	17 (63.0)	
**Patients with HCC relapse, n° (%)**	9 (32.1)	10 (37.0)	0.70
**Patients with a single HCC, n° (%)**	17 (60.7)	10 (37.0)	
**Patients with multiple HCC, n° (%)**	11 (39.3)	17 (63.0)	0.08
**Patients with portal thrombosis, n° (%)**	1 (3.6)	2 (7.4)	0.53
**BCLC score, n° (%) of patients with**			
- **A**	25 (89.3)	22 (81.5)	
- **B**	2 (7.1)	3 (11.1)	
- **C**	1 (3.6)	2 (7.4)	0.4

### Evaluation of miR-125a target genes

In a recent study, we have evaluated the ability of miR-125a to interfere with the expression of known target genes in hepatocellular carcinoma HepG2 cells. The microRNA was transfected into the cells and the expression levels of ERBB2, ERBB3, MMP11, Zbtb7a, SIRT7, and VEGF-A were measured 48 h later. This analysis showed significant downregulation of MMP11, Zbtb7a and SIRT7 whereas the other targets were unaffected [22]. In the same study, we found that c-Raf is a direct target of miR-125a. Based on these data collected *in vitro*, we measured the expression level of MMP11, Zbtb7a, SIRT7 and c-Raf in tumor biopsies, focusing on those patients showing a downregulation of miR-125a of at least 2-fold. Comparison with the adjacent NC tissue revealed that MMP11, SIRT7 and c-Raf were upregulated in 71-83% of the patients with mean fold regulation values of 3, 2.2 and 1.7, respectively (Table 3). On the other hand, Zbtb7a didn't show a significant upregulation.

**Table 3:** Expression levels of validated targets of miR-125a in hepatocellular carcinoma

		Fold regulation (HCC/NC)			
Etiology	Patient	MMP11	SIRT7	c-Raf	Zbtb7a
	1	n.d.	0.40	0.50	0.60
HBV	26	0.92	0.65	1.02	0.67
	30	5.07	1.85	0.95	1.41
	5	1.49	1.38	1.18	1,83
	32	1.61	2.51	1.53	0.05
	35	0.46	1.51	2.46	1.77
	36	4.61	4.20	2.47	2,29
HCV	39	n.d.	2.92	1.06	0.71
	43	3.44	2.42	1.99	0.54
	54	0.78	4.24	0.73	1.42
	56	9.97	1.95	5.35	0.36
	65	2.10	2.00	2.30	1.70
	23	2.50	n.d.	0.99	1.17
NASH	49	3.37	n.d.	1.53	0.70
	mean	3.03*	2.17*	1,72*	1.09

Expression levels were evaluated by RT-qPCR in a subset of patients with a downregulation of miR-125a of at least 2-fold. Data are reported as fold regulation for comparison of hepatocellular carcinoma (HCC) tissue with matched adjacent non-tumor liver tissue (NC). N.d., not determined because of the limited amount of tissue. * p < 0.05 at Wilcoxon test.

## DISCUSSION

MicroRNA-125a-5p, denominated lin-4 in nematodes, appeared early in evolution, no later than 550 millions of years ago. Today, it is present in all animals with bilateral symmetry and its nucleotide sequence is very well conserved, with an 11 nucleotide stretch encompassing the seed region that is identical in all species analyzed [24]. This microRNA plays a fundamental role in downregulating Lin-28 protein, thus promoting phase transitions in development and/or cell differentiation in nematodes, insects and mammals [36–39]. In mammalians, miR-125a appears to be expressed in most tissues, mainly targeting membrane receptors or intracellular signal transductors of mitogenic signals, thus limiting cell proliferation. Expression of this miRNA generally increases with cell differentiation whereas it is downregulated in several types of tumors, such as breast [25–27], lung [32, 33], ovarian [40], gastric [41], colon [42], and cervical [43] cancers, neuroblastoma [29], medulloblastoma [30], glioblastoma [31], and retinoblastoma [44]. Limited studies on the expression of miR-125a in HCC have also been performed. In 2013, Kim JK et al. used microarrays to analyze 16 samples of HCC of unknown etiology and compared the expression of the miRNA with 8 samples on non-matched liver tissue, showing a significant downregulation of miR-125a [34]. Similar results were obtained by Bi Q et al. that analyzed 80 samples of HCC, mostly from patients with chronic hepatitis B [35]. Then, our work is the first study conducted on a large cohort of well-characterized patients with chronic hepatitis C. It demonstrates a marked down-regulation of miR-125a in tumor tissue and a subsequent up-regulation of its oncogenic targets MMP11, SIRT7 and c-Raf. These data suggest an oncosuppressor effect of the microRNA on HCC of different etiologies, likely through the regulation of MMP11, c-Raf and SIRT7 expression. No correlation between the down-regulation of miR-125a and severity of HCC was observed, suggesting that the miRNA is mainly involved in the initiation of the oncogenic process. However, the majority of patients had an early stage of HCC, whereas this correlation should be evaluated in a larger sample of patients with HCC at different stages. The correlation between higher serum level of total bilirubin and higher down-regulation of miR-125a is intriguing. Recently, Han and colleagues evaluated the factors associated with the recurrence of HCC in 250 recipients of liver transplantation; they showed a correlation between lower serum values of total bilirubin in the donors and higher rate of HCC recurrence in the recipients, suggesting a protective role of bilirubin due to an anti-oxidant effect [45]. Thus, we may hypothesize that a higher down-regulation of miR-125a and a subsequent up-regulation of its targets may cause an activation of anti-oxidant factors, including serum bilirubin. However, this hypothesis should be confirmed in specific studies.

Besides their central role in control of cell proliferation and differentiation, microRNAs act as regulators of virus-host interactions [46, 47]. With regard to hepatotropic viruses, miR-125a has been shown to interfere with the expression of hepatitis B virus surface antigen [48–50] thus limiting viral replication [51]. It is not uncommon that a microRNA affects multiple cellular activities since a single miRNA can bind several mRNAs provided with the same target sequence. In the case of miR-125a, the antiproliferative activity toward hepatic cells and the antireplicative activity toward HBV are clearly due to inhibition of two different pathways but they may be functionally related. Since HBV has appeared late in evolution, infecting only mammals and birds, primary role of miR-125a is most likely fine tuning of cell proliferation. HBV may have then coopted this cellular miRNA to modulate its own replication, keeping it low to escape the immune system and establish a persistent infection. It may also be speculated that HBV has become sensitive to miR-125a, among hundreds of other hepatic miRNAs, to coordinate its replication to the host cell proliferation.

Downregulation of microRNAs in cancer cells may be determined by genetic or epigenetic factors [52]. Chromosomal abnormalities, deletions, and mutation of promoter regions can reduce the expression level of miRNAs [53, 54]. It is also known that several miRNA genes are associated to CpG islands and are downregulated by DNA methylation [55]. With regard to miR-125a, a recent study has shown its downregulation in acute myeloid leukemic cells due to aberrant methylation of a CpG island located 3544 bp upstream of the mature miRNA sequence [56]. Further investigations aimed at the identification of the promoter of the transcription unit of miR-125a will allow the comprehension of the molecular mechanisms governing its expression, eventually leading to treatments to restore its expression in tumor cells. Otherwise, vectors for the ectopic expression of miRNA mimics [57] may be used to boost the cellular reservoir of miR-125a. Finally, recent progress in cell transfection techniques has led to development of efficient formulations for therapeutic delivery of synthetic microRNAs based on cationic polymers or exosomes [58, 59]. These techniques have made the use of small RNAs in human therapy a promising therapeutic approach and several clinical trials are in progress [60].

## PATIENTS AND METHODS

### Patients

This study was planned as prospective with a progressive enrolment by the senior investigators of two participating Liver Units in Naples, southern Italy. The two centers participating to the study have cooperated in several investigations using the same clinical approach [61]. All consecutive patients who underwent a diagnostic liver biopsy for HCC at one of two participating Liver Units from June 2013 to May 2014 were enrolled. HCC was diagnosed in accordance with the EASL/EORTC criteria [62]. Each patient underwent a complete physical examination, full liver function tests and serology for HBsAg, anti-delta, anti-HIV and anti-HCV. The anti-HCV-positive subjects were considered as having HCV infection. A diagnosis of NASH (non-alcoholic steato-hepatitis) was made according to the AASLD/ACG/AGA guidelines [63]. Alcohol intake and other potential causes of liver disease were assessed. A consumption of alcohol exceeding 30g per day for females and 40g per day for males over at least the last 6 months was considered as alcohol abuse. None of the patients was anti-HIV positive.

The stage of HCC was assessed using the criteria proposed by the BCLC (Barcelona Clinic Liver Cancer) group [64]. For each patient, a HCC specimen and a non-neoplastic liver tissue sample (NC) were obtained by US-guided percutaneous liver biopsy using a needle Biomol® 18Gx150mm (HS Hospital, Rome, Italy). Fragments of nearly 3 mg were cut away from the two extremities of the liver biopsies not useful for diagnosis [65] and stored at -80°C in RNAlater solution (Qiagen GmbH, Hilden, Germany) for subsequent molecular analyses. In addition, plasma and whole blood samples were collected for each patient and stored at -80°C the same day the liver biopsies were performed. All procedures were followed in accordance with the international guidelines and with the Helsinki Declaration of 1975, revised in 1983. The Ethics Committee of the Azienda Ospedaliera Universitaria of the Second University of Naples approved the study (n°349/2013). All patients signed their informed consent for liver biopsy, the collection and storage of biological samples and for the anonymous use of their data for research purposes.

### Sero-virological methods

HBV serum markers (HBsAg, anti-HBs, anti-HBc) were sought using commercial immunoenzymatic assays (Abbott Laboratories, North Chicago,IL, USA). The anti-HCV antibody was detected using a 3rd generation commercial immunoenzymatic assay (Ortho Diagnostic Systems, Neckargemund, Germany). Liver biochemistry and routine analyses were performed by standard methods in a Cobas Modular 6,000 automated analyzer using c501 biochemistry modules (Roche Diagnostics Ltd, Rotkreuz, Switzerland). For HBsAg positive patients, serum HBV-DNA levels were determined by real-time PCR with a detection limit of 20 copies/mL, as previously described [66]. For anti-HCV positive patients, viral RNA was extracted from 140 μl of plasma samples using a microspin column (QIAamp RNA viral kit, Qiagen GmbH). HCV RNA was quantified by real-time PCR in a Light cycler 1.5 (Roche Diagnostics, Branchburg, NJ, USA), as reported [67]; the detection limit of this method is about 40 IU/mL of plasma. HCV genotypes were determined by the HCV genotype Lipa assay (Bayer, France) following the manufacturer's instructions.

### Tissue RNA extraction and real-time qPCR analyses

HCC and NC liver tissues were homogenized by TissueLyser (QiagenGmbH, Hilden, Germany) at 30 Hz for 30 sec. Total RNA was then extracted by microspin columns (AllPrep DNA/RNA mini kit, Qiagen GmbH, Hilden, Germany) and quantitated spectrophotometrically with NanoDrop 2000c (ThermoScientific). MiR-125a was quantified along with RNU6B (reference gene) by RT-qPCR with TaqMan® miRNA assays from Applied Biosystems according to the manufacturer's protocol. The expression levels of transcripts targeted by miR-125a were determined by RT-qPCR with iTaq™ Universal SYBR^®^ Green Supermix (Bio-Rad). In particular, 200 ng of RNA were retrotranscribed by Transcriptor High Fidelity cDNASynthesis Sample kit (Roche) using random examer primers; 1 μl of cDNA product was then used to amplify the target sequences along with GAPDH as reference. Primers were: GAPDH, 5′-GAAGGTGAAGGTCGGAGTC-3′ and 5′-GAAGATGGTGATGGGATTT-3′; SIRT7, 5′-GTCTG CATGAGCAGAAGCTG-3′ and 5′-GGAACGCAGGA GGTACAGAC-3′; c-Raf, 5′-GCAATGAAGAGGCTGGT AGC-3′ and 5′- GGAGCAGCTCAATGGAAGAC-3′; Zbtb7a, 5′-ACGAGTGCAACATCTGCAAG-3′ and 5′-GG TCGTAGTTGTGGGCAAAG-3′ [33]; MMP11, 5′-TCC TGACTTCTTTGGCTGTG-3′ and 5′-CCATGGGTCT CTAGCCTGAT-3′ [35]. The expression levels of miR-125a and its targeted transcripts were normalized to their respective reference genes by using the 2^−ΔCt^ method.

### Statistical analysis

Continuous variables were summarized as mean ± standard deviation, unless stated differently; categorical variables were expressed as absolute and relative frequencies. Differences in the mean values were evaluated by the Student's t-test except for the expression levels of miR-125a targets that did not follow a normal distribution and were compared by the Wilcoxon test; the chi-squared test was used to compare categorical variables. A p value <0.05 was considered to be statistically significant.

## Abbreviations

HCC: hepatocellular carcinoma
NC: non-neoplastic liver tissue
miRNA: microRNA
miR-125a: human miR-125a-5p
SIRT7: sirtuin-7
VEGF-A: vascular endothelial growth factor A
MMP11: matrix metalloproteinase-11
HBV: hepatitis B virus
HCV: hepatitis C virus
NASH: non-alcoholic steatotic hepatitis
